# Atlanta Medical College

**Published:** 1895-04

**Authors:** 


### Editorial.

### ATLANTA MEDICAL COLLEGE.

On the evening of April 2nd, a large and appreciative audience gathered at the Grand Opera House to witness the thirty-seventh annual commencement exercises of this time-honored institution. The exercises were opened with prayer by Rev. Dr. Henry McDonald. After this the report of the Proctor, Dr. W. S. Kendrick, was read as follows:

### THE PROCTOR'S REPORT.

Dr. Kendrick's report was full of interest and showed that the college has grown and prospered during the last year in spite of the hard times that have prevailed on all sides. It was as follows:

Through peace and war, sunshine and storm, the Atlanta Medical College has lived and thrived.

She built and builded well, and this commencement evening is the crowning glory of all her past achievements.

With an able and harmonious faculty, with a building well-equipped and with students representing twenty-two States, unparallelled success has come to the institution over which you preside. The urgent demands for higher medical education prompted us one year ago to adopt a Three years' course of six months each.

Occupying this elevated plane, she is in position to compete with the strongest, and in the competition maintain her great popularity and exalted standing. In the various departments Georgia has furnished 223; Alabama, 35; Texas, 19; Mississippi, 19; South Carolina, 15; Florida, 14; North Carolina, 14; Lousiana, 11; Tennessee 8; West Virginia, 5; Arkansas, 3; Kentucky, 3; New York, 3; Indiana, 2; Pennsylvania, 1; Virginia, 1; India, 1; California, 1; Missouri, 1; Indian Territory, 1; Illinois, 1; Nova Scotia, 1; and Canada, 1; a total of 383.

Of these, 135 in the medical class ask for the degree of Doctor of Medicine, and four in the Pharmacuetical, for the degree of graduate in pharmacy. Justice demands that of this class it should be said, in deportment, application and earnest effort, it has excelled many of its predecessors. Proud of their victory, happy in the realization of their hopes, and standing in the dawning of their first professional day, they are now ready to receive your official recognition.

The degrees were then conferred in Latin by Col. N. J. Hammond, President of the Board of Trustees. This added much to the dignity of the occasion.

Prof. H. C. White, of Athens, orator of the evening, made a splendid address replete with sound advice.

Dr. J. C. King, of Lousiana, valedictorian, made a good address and did ample justice to the occasion.

The prizes were delivered by Col. Hammond, to the following gentlemen:

Dr. J. W. Torbett, of Texas, was awarded the first honor, and it was a signal honor as he made the highest average ever made by any student in the college.

The second honor was shared by Dr. E. C. Cartledge, of Georgia, and Dr. H. McCullough, of Alabama, while the third honor was shared by Dr. W. J. Shaw and Dr. C. L. Youmans.

The prizes were delivered by Col. Hammond with his usual grace and eloquence.

Dr. J. R. Murray, Dr. T. C. Bair, Dr. C. W. Westmoreland, Dr. G. W. Herriot, Dr. G. D. Dorrough, Dr. E. L. Green, Dr. J. G. Bouvier, Dr. C. B. M. Woods, Dr. J. D. W. Brannan, Dr. H. B. Ross, Dr. B. C. Daniel, Dr. W. H. Alexander and Dr. H. L. Bauer received honorable mention for the stand they had taken in their classes during the year.

The exercises were interesting from first to last and were greatly enjoyed by all present.

